# Supplementary material for: Effect of Solvents, Stabilizers and the Concentration of Stabilizers on the Physical Properties of Poly(d,l-lactide-co-glycolide) Nanoparticles: Encapsulation, In Vitro Release of Indomethacin and Cytotoxicity against HepG2-Cell
Source: Pharmaceutics. 2022 Apr 15;14(4):870. doi: 10.3390/pharmaceutics14040870 (PMC9028368; doi:10.3390/pharmaceutics14040870)

Effect of solvents, stabilizers and concentration of stabilizers on the physical properties of Poly (D, L-lactide-co-glycolide) nanoparticles: Encapsulation, *in vitro* release of Indomethacin and cytotoxicity against HepG2-cell

Musaed Alkholief [1], Mohd Abul Kalam [1], Md Khalid Anwer [2], Aws Alshamsan [1, *]

[1]*Nanobiotechnology Unit, Department of Pharmaceutics, College of Pharmacy, King Saud University, PO Box-2457, Riyadh-11451, Saudi Arabia;* malkholief@ksu.edu.sa *(MA),* makalam@ksu.edu.sa *(MAK)*

[2]*Department of Pharmaceutics, College of Pharmacy, Prince Sattam Bin Abdulaziz University, Al-Kharj-11942, Saudi Arabia;* m.anwer@psau.edu.sa *(KA)*

Corresponding author email ID: aalshamsan@ksu.edu.sa (Prof. Aws Alshamsan)

Figure S1. Different kinetic model plots for PLGA-NPs-1 (prepared with 1%, w/v PVA as stabilizer and $CHCl_3$ as organic solvent) (A) and PLGA-NPs-2 (prepared with 3%, w/v PVA as stabilizer and $CHCl_3$ as organic solvent (B)

Figure S2. Different kinetic model plots for PLGA-NPs-5 (prepared with 1%, w/v PVA as stabilizer and DCM as organic solvent) (A) and PLGA-NPs-6 (prepared with 3%, w/v PVA as stabilizer and DCM as organic solvent (B)

Figure S3. Different kinetic model plots for PLGA-NPs-9 (prepared with 1%, w/v PVP as stabilizer and $CHCl_3$ as organic solvent) (A) and PLGA-NPs-10 (prepared with 3%, w/v PVP as stabilizer and $CHCl_3$ as organic solvent (B)

Figure S4. Different kinetic model plots for PLGA-NPs-13 (prepared with 1%, w/v PVP as stabilizer and DCM as organic solvent) (A) and PLGA-NPs-14 (prepared with 3%, w/v PVP as stabilizer and DCM as organic solvent (B)

Figure S1

(A) 1%, w/v PVA as stabilizer and $CHCl_3$ as organic solvent

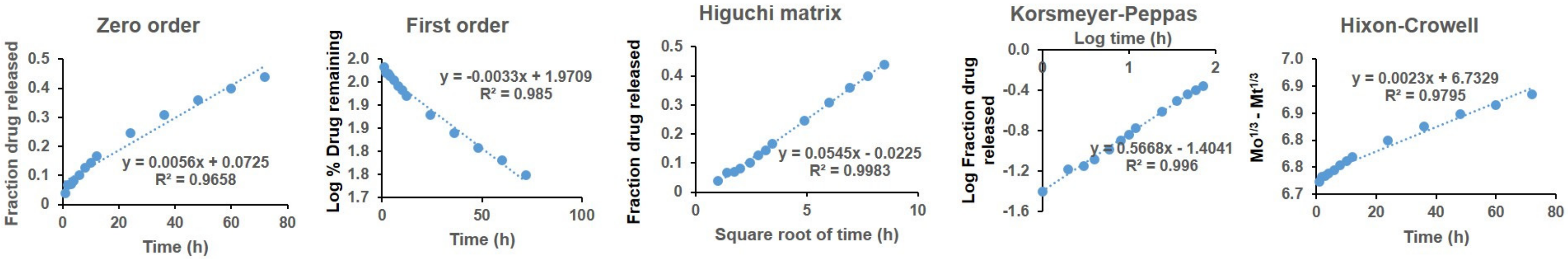

(B) 3%, w/v PVA as stabilizer and $CHCl_3$ as organic solvent

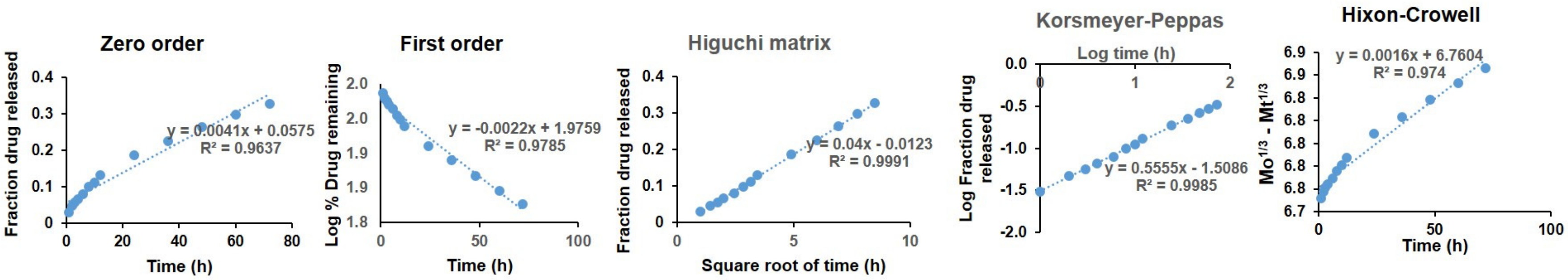

Figure S2

(A) 1%, w/v PVA as stabilizer and DCM as organic solvent

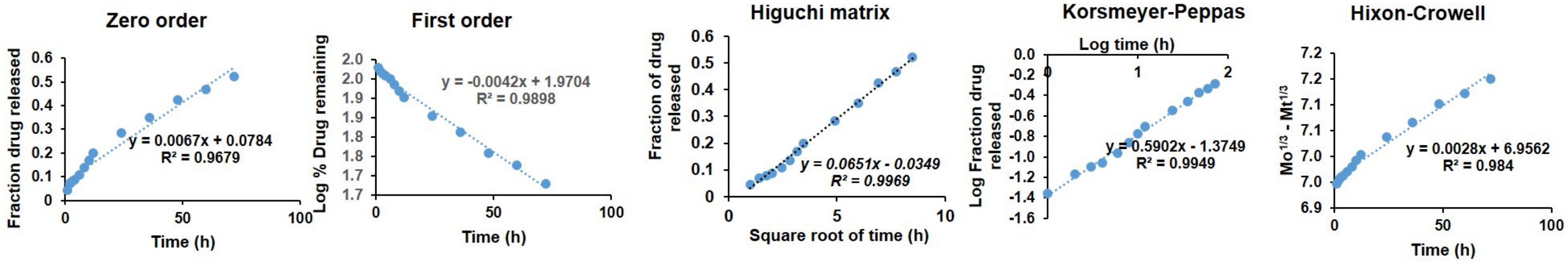

(B) 3%, w/v PVA as stabilizer and DCM as organic solvent

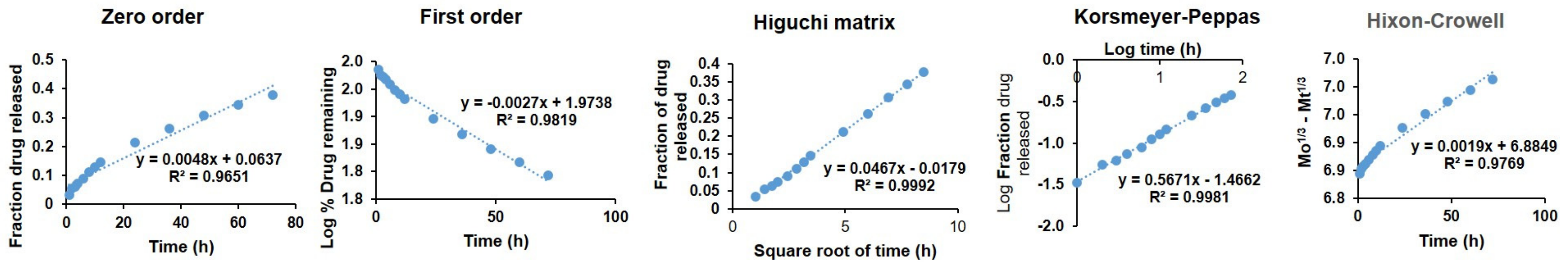

Figure S3

(A) 1%, w/v PVP as stabilizer and $CHCl_3$ as organic solvent

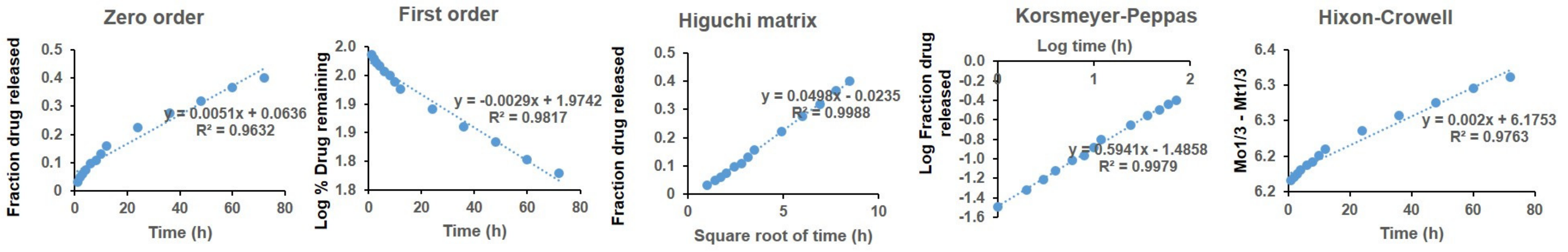

(B) 3%, w/v PVP as stabilizer and $CHCl_3$ as organic solvent

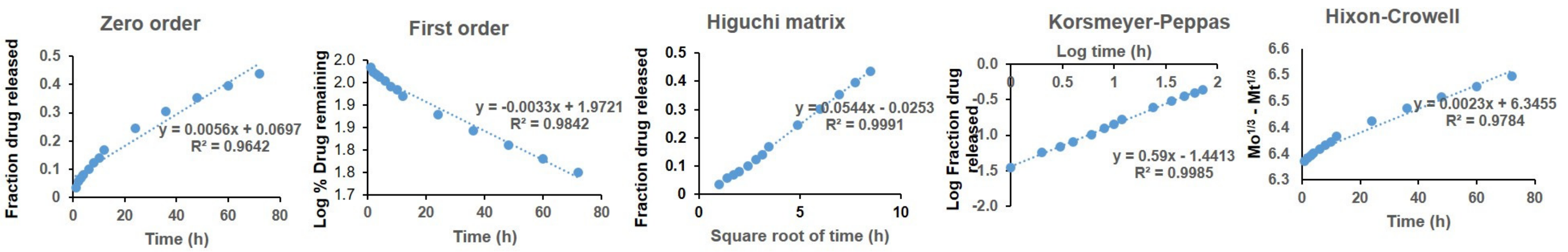

Figure S4

(A) 1%, w/v PVP as stabilizer and DCM as organic solvent

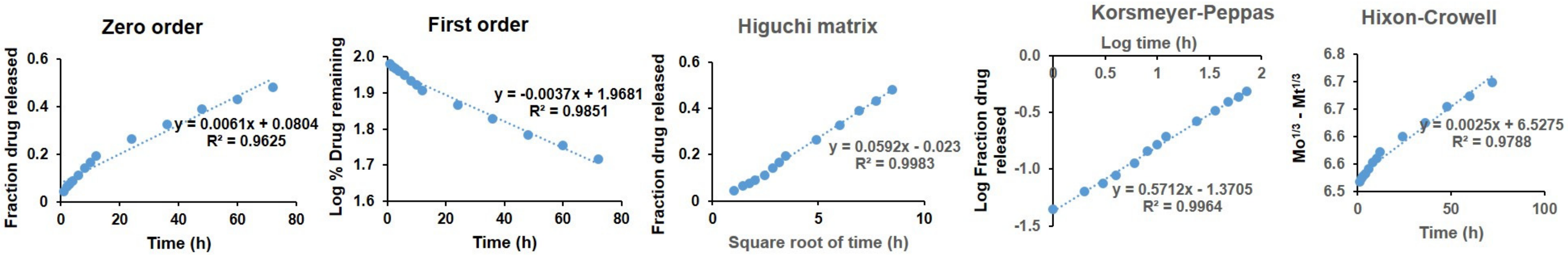

(B) 3%, w/v PVP as stabilizer and DCM as organic solvent

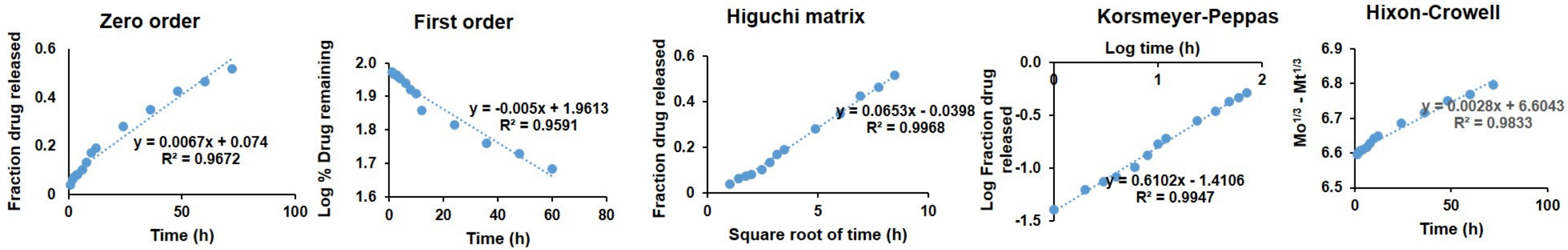

Supplement: Supplementary file 1 [file pharmaceutics-14-00870-s001.zip › pharmaceutics-1607091-supplementary.pdf]
